# Karyotypes of some medium-sized Dytiscidae (Agabinae and Colymbetinae) (Coleoptera)

**DOI:** 10.3897/CompCytogen.v7i2.5223

**Published:** 2013-06-13

**Authors:** Robert B. Angus, Molly J. Clery, Jodie C. Carter, Daniel E. Wenczek

**Affiliations:** 1 School of Biological Sciences, Royal Holloway, University of London, Egham Hill, Egham, Surrey TW20 0EX, UK; 2 Department of Life Sciences (Entomology), The Natural History Museum, Cromwell Road, London SW7 5BD, UK

**Keywords:** Chromosomes, karyotypes, sex chromosome systems, Dytiscidae, *Agabus*, *Colymbetes*, *Rhantus*

## Abstract

An account is given of the karyotypes of 29 species of medium sized Dytiscidae (Coleoptera). Of the 20 species of *Agabus* Leach, 1817, 18 have karyotypes comprising 21 pairs of autosomes and sex chromosomes which are either X0(♂) or XX (♀). These species are *Agabus serricornis* (Paykull, 1799), *Agabus labiatus* (Brahm, 1791), *Agabus congener* (Thunberg, 1794), *Agabus lapponicus* (Thomson, 1867), *Agabus thomsoni* (J. Sahlberg, 1871), *Agabus confinis* (Gyllenhal, 1808), *Agabus sturmii* (Gyllenhal, 1808), *Agabus bipustulatus* (Linnaeus, 1767), *Agabus nevadensis* Håkan Lindberg, 1939, *Agabus wollastoni* Sharp, 1882, *Agabus melanarius* Aubé, 1837, *Agabus biguttatus* (Olivier, 1795), *Agabus binotatus* Aubé, 1837, *Agabus affinis* (Paykull, 1798), *Agabus unguicularis* (Thomson, 1867), *Agabus ramblae* Millan & Ribera, 2001, *Agabus conspersus* (Marsham, 1802) and *Agabus nebulosus* (Forster, 1771). However two species, *Agabus infuscatus* Aubé, 1838 and *Agabus adpressus* Aubé, 1837, have developed a neo-XY system, with karyotypes comprising 21 pairs of autosomes and XY sex chromosomes (♂). No chromosomal differences have been detected between typical *Agabus bipustulatus* and *Agabus bipustulatus* var. *solieri* Aubé, 1837, nor have any been found between the three species of the *Agabus bipustulatus* complex (*Agabus bipustulatus*, *Agabus nevadensis* and *Agabus wollastoni*). The four species of *Colymbetes* Clairville, 1806, *Colymbetes fuscus* (Linnaeus, 1758), *Colymbetes paykulli* Erichson, 1837, *Colymbetes piceus* Klug, 1834 and *Colymbetes striatus* (Linnaeus, 1758) have karyotypes comprising 20 pairs of autosomes and sex chromosomes which are X0 (♂), XX (♀). Two of the species of *Rhantus* Dejean, 1833, *Rhantus exsoletus* (Forster, 1771) and *Rhantus suturellus* (Harris, 1828) have karyotypes comprising 20 pairs of autosomes and X0/XX sex chromosomes, but the other three species, *Rhantus grapii* (Gyllenhal, 1808), *Rhantus frontalis* (Marsham, 1802) and *Rhantus suturalis* (Macleay, 1825) have 22 pairs of autosomes and X0/XX sex chromosomes. *Agabus congener* and *Rhantus suturellus* may have one B-chromosome. Nine of the species have previously published karyotype data but for seven of these the data are wrong and are here corrected.

## Introduction

When Smith and Virkki (1976) compiled their list of beetles whose chromosome numbers were known, they gave data for 2120 species, including 138 named species belonging to the suborder Adephaga. Of these 110 were Carabidae, 21 Dytiscidae and 7 Gyrinidae. By 1984 the number of carabid species whose chromosome numbers were known had increased to 426 (Serrano and Yadav 1984) and the number of Dytiscidae had reached 32, though five of these were unidentified (Yadav et al. 1984). Interestingly, the total number of world species of Carabidae is given as “more than 40,000” (Wikipedia) while the number for Dytiscidae is about 4080 (Nilsson-Örtmann and Nilsson 2010), so at this stage the proportion of species for which chromosome numbers are listed in the two families is about the same. Data have continued to accumulate, so that Galian and Moore (1994) give the number of carabid species whose chromosome numbers are known as “more than 800”. Numbers for Dytiscidae have also continued to increase. Saleh Ahmed et al. (2000) gave data on 1 species of *Hydrovatus* Motschulsky, 1 *Hydroporus* Clairville and 3 *Nebrioporus* Régimbart (Hydroporinae), 1 *Agabus* Leach (Agabinae), 1 *Colymbetes* Clairville (Colymbetinae) and 1 *Eretes* Laporte and 1 *Hydaticus* Leach (Dytiscinae). Aradottir and Angus (2004) gave information on 7 species of *Ilybius* Erichson (Agabinae), Dutton and Angus (2007) described the karyotypes of 7 species of the “*Stictotarsus griseostriatus* (De Geer) group” (now in the genus *Boreonectes* Angus) (Hydroporinae), and Tatton and Angus (2011) reported on 30 species related to *Deronectes* Sharp (Hydroporinae), of which 27 had no previously published data, bringing to total number of dytiscid species with known chromosome numbers to about 82. This gives both the Carabidae and the Dytiscidae as having about 2% of their species with known chromosome numbers.

The present paper reports on 20 *Agabus* species, of which only four had previously published chromosome data (wrong for three of the species), 4 *Colymbetes*, all of which have previously published data, though for three of the species these data were wrong, and 5 *Rhantus* of which one species had published data, again wrong. This gives a net increase to over 100 in the number of dytiscid species for which information on chromosome numbers are available. The data have been gathered over more than 25 years, and include the results of research projects by three undergraduate students of Royal Holloway, University of London, supervised by R. B. Angus. D. E. Wenczek (1994) studied *Rhantus* Dejean, J. C. Carter (2001) *Rhantus* and *Colymbetes*, and M. J. Clery (2009) made a special study of the *Agabus bipustulatus* (Linnaeus) species group.

## Material and Methods

The species studied, with their localities of origin, collectors and dates, as well as the number of specimens yielding successful preparations, is given in Table 1. Nomenclature and classification follow Nilsson and Hájek (2013, internet version). Where there is more than one locality for a given species the localities from which various preparations came are given in the figure captions. Otherwise localities are not given apart from in the table.

**Figure 1.**
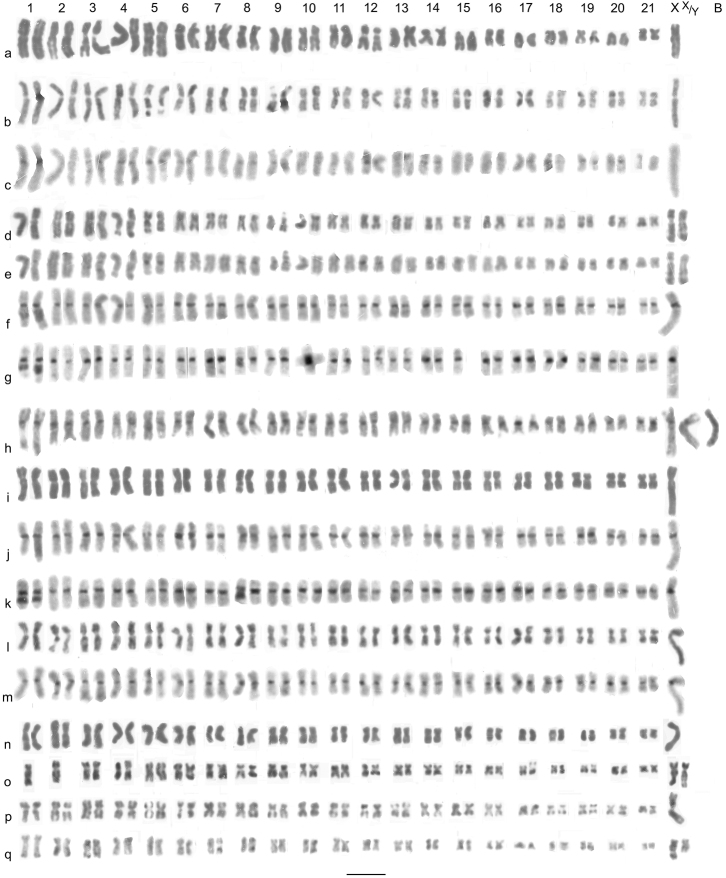
*Agabus s. str*. (**a–e**) and *Agabus (Acatodes)* (**f**– **q**), mitotic chromosomes arranged as karyotypes. **a** *Agabus serricornis*, ♂, mid-gut, plain **b**, **c** *Agabus labiatus*, ♂, mid-gut **b** plain **c** C-banded **d**, **e** *Agabus labiatus*, ♀, mid-gut **d** plain, **e** C-banded **f**, **g** *Agabus congener*, ♂, Scotland, testis, C-banded **h** *Agabus congener*, ♀, Sweden, mid-gut, C-banded, with 1 B-chromosome **i**– **k** *Agabus lapponicus*, ♂, Sweden, testis **i** plain **j**, **k** C-banded **l**, **m** *Agabus thomsoni*, ♂, mid-gut **l** plain **m** the same nucleus C-banded **n**, **o** *Agabus confinis*, ♀, mid-gut, plain **n** lacking one X chromosome **o** lacking one replicate each of autosomes 1 and 2 **p** *Agabus sturmii*, ♂, mid-gut, plain **q** *Agabus infuscatus*, ♂, mid-gut, plain. Bar = 5μm.

Preparations were made from adult beetles, using mid-gut, testis and ovary, following the protocol given by Shaarawi and Angus (1991) and Dutton and Angus (2007). Treatment with colchicine and hypotonic KCl was for 12.5 min in each solution. C-banding was obtained using saturated Ba(OH)_2_ at room temperature, followed by incubation in salt-sodium citrate (2 X SSC) at 60° C. Treatment times varied, and the technique evolved over the more than 25 years of the study. If a treatment has been insufficient to produce C-banding, it may be repeated. Initially Angus used to clear the stain with a short immersion in 2X SSC at 60° C, but later found this unnecessary. One set of early experiments with *Agabus congener* and *Agabus lapponicus* was particularly interesting: an initial treatment of 5 min in Ba(OH)_2_ proved inadequate. A repeat treatment with 5 min in Ba(OH)_2_ produced good centromeric C-bands, but if the second treatment was for 3 min the secondary constrictions were also stained (Fig. 1f, g, and k with the secondary constrictions, Fig. 1j with just the centromeric C-bands).

**Table 1.** Material studied.

**Species**	**Locality**	**Collector, date**	**Material**
Genus *Agabus* Leach, 1817			
Subgenus *Agabus* s. str.			
*Agabus serricornis* (Paykull, 1799)	SWEDEN: Västerbotten, Åmsele.	A. N. Nilsson, 1990	1♂
*Agabus labiatus* (Brahm, 1791)	FINLAND: Lapponia Inarensis, Inari	R. B. Angus, 2008	1♂, 1♀
Subgenus *Acatodes* C. G. Thomson, 1859			
*Agabus congener* (Thunberg, 1794)	SCOTLAND: Ayrshire, Knockewart Moss	G. N. Foster, 1986	2♂♂, 1♀
	SWEDEN: Västerbotten, Sirapsbaken	A. N. Nilsson, 1986	2♂♂, 1♀
*Agabus lapponicus* (Thomson, 1867)	SWEDEN: Västerbotten, Skörträskberget	A. N. Nilsson, 1986	3♂♂, 1♀
*Agabus thomsoni* <br/>(J. Sahlberg, 1871)	NORWAY: Finnmark east, Bugøynes	R. B. Angus, 2008	1♂
*Agabus confinis* (Gyllenhal, 1808)	SWEDEN: Västerbotten, Vindeln, Strycksele	A. N. Nilsson, 1991	3♀♀
*Agabus sturmii* (Gyllenhal, 1808)	ENGLAND: Surrey, Chobham Common	R. B. Angus, 1991	1♂, 1♀
*Agabus infuscatus* Aubé, 1838	NORWAY: Finnmark east, Bugøynes	R. B. Angus, 2008	1 ♂
Subgenus *Gaurodytes* C. G. Thomson, 1859			
*Agabus bipustulatus* (Linnaeus, 1767)	ENGLAND: Surrey, Wisley Common	R. B. Angus & M. J. Clery, 2008	3♂♂
	Hampshire, Woolmer Bog	R. B. Angus, 2008	3♂♂,1 ♀
	Worcestershire, Wyre Forest	R. B. Angus & M. J. Clery, 2008	3 ♂♂
	FINLAND: Lapponia Inarensis, Inari	R. B. Angus, 2008	1 ♂
	SWEDEN: Norbotten, near Umeå	M. Drotz, 1996	1 ♂
*Agabus bipustulatus* var. *solieri* Aubé, 1837	SWITZERLAND, Valais, small lake S of Illsee	R. B. Angus, 2008	3♂♂, 1 ♀
	Valais, ditch near the Moiry glacier	R. B. Angus, 2008	2 ♂♂
	FRANCE: Hautes-Alpes, Guillestre	M. Drotz, 1998	2♂♂
*Agabus nevadensis* Håkan Lindberg, 1939	SPAIN: Granada, Sierra Nevada	M. Drotz, 1999	1 ♂, 1 ♀
*Agabus wollastoni* Sharp, 1882	MADEIRA: Pico Arieño	A. N. Nilsson, 1998	2 ♂♂, 1 ♀
*Agabus melanarius* Aubé, 1837	ENGLAND: East Sussex, Hindleap Warren	R. B. Angus & M. J. Clery, 2008	1 ♂, 1 ♀
*Agabus biguttatus* (Olivier, 1795)	EGYPT (Saleh Ahmed et al., 2000): El Noqra	R. Saleh Ahmed & R. B. Angus, 1994	1 ♂
	SARDINIA: Medio Campidano, Giara di Gesturi	R. B. Angus, 1994	1♂
*Agabus binotatus* Aubé, 1837	CORSICA: Corse-du-Sud, Col de Vizzavona.	R. B. Angus, 1993	1 ♂
*Agabus affinis* (Paykull, 1798)	ENGLAND: Hampshire, New Forest	R. B. Angus, 1987	1 ♂
*Agabus unguicularis* (Thomson, 1867)	ENGLAND: Norfolk, East Walton Common	R. B. Angus, 1987	2 ♂♂
*Agabus ramblae* Millan & Ribera, 2001	SPAIN: Huesca, Villanueva de Sigena, Barranco del Hospital	I. Ribera, G.N. Foster, D. Lott & P. Aguilera, 1995	2♂♂
	Murcia, Rambla de Majada en El Pilón	A. Millan, 1995	1 ♀
*Agabus conspersus* (Marsham, 1802)	ENGLAND: Hampshire, Keyhaven	R. B. Angus, 1993	1 ♂
*Agabus nebulosus* (Forster, 1771)	ENGLAND: East Sussex, Cuckmere Haven	R. B. Angus, 1993	1♂
	CANARY ISLANDS: Tenerife	A. N. Nilsson, 1994	1♂, 2 ♀♀
*Agabus adpressus* Aubé, 1837	NORWAY: Finnmark east, Bugøynes	R.B. Angus, 2008	1 ♂
Genus *Colymbetes* Clairville, 1806			
*Colymbetes fuscus* (Linnaeus, 1758)	ENGLAND: Surrey, Wisley Common	R. B. Angus, 2000	1 ♂
	FRANCE: Indre, Pinail	R. B. Angus, 2000	1 ♂
*Colymbetes paykulli* Erichson, 1837	SWEDEN: Ångermanland, Hörnsjö, lake Uthörnsjön	A. N. Nilsson, 2000	1 ♂
	Ångermanland, Mullsjö	A. N. Nilsson, 2000	1 ♂
*Colymbetes piceus* Klug, 1834	Egypt (Saleh Ahmed et al., 2000): El Noqra	R. Saleh Ahmed & R. B. Angus, 1994	1 ♂
*Colymbetes striatus* (Linnaeus, 1758)	SWEDEN: Ångermanland, Hörnsjö, lake Uthörnsjön	A. N. Nilsson, 2000	1 ♂
Genus *Rhantus* Dejean, 1833			
Subgenus *Nartus* Zaitsev, 1907			
*Rhantus grapii* (Gyllenhal, 1808)	ENGLAND: Dorset, Studland Heath	R. B. Angus, 1993	2♂♂
Subgenus *Rhantus* s. str.			
*Rhantus exsoletus* (Forster, 1771)	ENGLAND: Dorset, Studland Heath	R. B. Angus, 1993	1 ♂
	Norfolk, Gayton Thorpe Common	R. B. Angus, 1993	1 ♂
*Rhantus frontalis* (Marsham, 1802)	ENGLAND: Norfolk, Gayton Thorpe Common	R. B. Angus, 1993	1 ♂
	Norfolk, Thompson Common	R. B. Angus, 1993	1 ♂
*Rhantus suturalis* (Macleay, 1825)	ENGLAND: Dorset, Studland Heath	R. B. Angus, 2000	1 ♂
	Middlesex, Staines Moor	R. B. Angus, 2000	1 ♂
	KUWAIT: Ras Az Zawr	R. B. Angus, 1996	1 ♂
*Rhantus suturellus* (Harris, 1828)	FRANCE: Indre, Pinail	R. B. Angus, 2000	1 ♂
	ENGLAND: Dorset, Studland Heath	R. B. Angus, 1993, 2000	1♂, 2 ♀♀

Chromosome measurements were made on screen and were used for calculating Relative Chromosome Length (RCL), the length of each chromosome expressed as a percentage of the total haploid autosome length in the nucleus. This compensates for differing degrees of chromosome contraction shown in different nuclei. For the *Agabus bipustulatus* group the RCL data were subjected to statistical analysis using Student’s t-test, but otherwise they are given as approximate values only, to indicate the size relationships of the different pairs of autosomes. Centromere Indices (CI) are not given in detail, but are assigned to their conventional categories. Based on Sumner (2003) the categories are: metacentric–CI 46–50; submetacentric–CI 26–45; subacrocentric–CI 16–25; acrocentric–CI 3–15.

## Results

### Agabinae Thomson, 1867 *Agabus* Leach, 1817 Subgenus *Agabus* s. str.

*Agabus serricornis* (Paykull, 1799). Fig. 1a. Published information: none. 2n = 42 + X0 (♂). The RCLs of the autosomes range from about 7.6–2.5, with sharp decreases between pairs 5 (RCL about 6.4) and 6 (RCL about 5), 15 (RCL about 4.5) and 16 (RCL about 3.4), and 20 (RCL about 3.1) and 21 (RCL about 2.5). The X chromosome (RCL about 6.4) is similar in size to pairs 4 and 5. Most of the chromosomes are metacentric to submetacentric, with pairs 8–11 subacrocentric and pairs 15 and 20 more or less acrocentric. Pair 12 has a distinct secondary constriction at the base of its short arm. The X chromosome is subacrocentric, with the centromere clearly nearer the end than in autosomes 4 and 5. No C-banded material is available.

*Agabus labiatus* (Brahm, 1791). Fig. 1b, c (♂), Fig. 1d, e (♀). Published information: none. 2 n = 42 + X0 (♂), 42 + XX (♀). The autosomes, all either metacentric or submetacentric, have RCLs ranging from about 7.8–2.7, with a fairly gradual decrease along the karyotype, though this is slightly sharper between pairs 5 (RCL about 7.1) and 6 (RCL about 5.9) and 11 (RCL about 4.3) and 12 (RCL about 3.8). The X chromosome is submetacentric and the largest in the nucleus (RCL about 9). Pair 5 have secondary constrictions on the long arm and pair 13 on the short arm. C-banding (Fig. 1c, e) shows a limited development of centromeric C-bands. These are present on autosomes 1, 3–6, 12, 14 and 17–20. The remaining autosomes, and the X chromosome, lack C-bands. Many of the C-bands are very weak, with the strongest bands present on autosomes 5 and 12.

### Subgenus *Acatodes* C. G. Thomson, 1859

*Agabus congener* (Thunberg, 1794). Fig. 1f, g (♂), Fig. 1h (♀). Published information: none. 2 n = 42 + X0 (♂), 42 + XX (♀), 1 B-chromosome. The autosomes, all more or less metacentric, have RCLs ranging from about 7–4, with an even size decrease along the karyotype. The submetacentric X chromosome, RCL about 9, is clearly the longest in the nucleus. All the chromosomes have distinct centromeric C-bands, with some variation in strength between pairs, and autosomes 1 and 8 have secondary constrictions which may C-band, especially that on autosome 1. The C-banding reaction of the secondary constriction of autosome 8 is less pronounced, and the constriction may be apparent in only one of the replicates. The Swedish female (Fig. 1h) has one B-chromosome, about as long as autosome 1 and appearing uniformly partly heterochromatic.

*Agabus lapponicus* (Thomson, 1867). Fig. 1i–k (♂). Published information: none. 2n = 42 + X0 (♂). The karyotype of this species appears indistinguishable from that of *Agabus congener*.

*Agabus thomsoni* (J. Sahlberg, 1871). Fig. 1l, m (♂). Published information: none. 2n = 42 + X0 (♂). The karyotype of this species is very similar to those of *Agabus congener* and *Agabus lapponicus*, but the longest autosome with a secondary constriction is placed as no. 2 as in this material it appears distinctly shorter than the longest autosome (pair 1). It is possible that additional material would show this not to be the case. As in the preceding two species, the secondary constriction on autosome 8 is more conspicuous in one of the replicates.

*Agabus confinis* (Gyllenhal, 1808). Fig. 1n, o (♀). Published information: 2n = 40 + “XY” (sex chromosomes not identified) (Smith 1953). 2n = 44 (♀), probably 42 + XX. The material available for study was three females, and although no intact chromosomal complement was obtained, the 43 chromosomes shown in Fig. 1n exceed the number given by Smith. The suggestion that the X chromosome is the largest in the nucleus is based on comparison with the karyotypes of the three preceding species, all, like *Agabus confinis*, members of the *Agabus congener* group. In the interpretation given here, Fig. 1n lacks one X chromosome while Fig. 1o, from a different specimen, has both X chromosomes but lacks one replicate each of autosomes 1 and 2.

*Agabus sturmii* (Gyllenhal, 1808). Fig. 1p (♂). Published information: 2n = 40 + Xy_p_ (Suortti 1971). 2n = 42 + X0 (♂), 42 + XX (♀). The autosomes, all either metacentric or submetacentric, have RCLs ranging from about 6.8–2.7. There is a fairly even decrease in length to pair 16 (RCL about 4.8), then a more abrupt decrease to pairs 17–20 (RCL about 3.4) and a further drop to pair 21 (RCL about 2.7). The X metacentric chromosome, RCL about 11.5, is by far the longest in the nucleus, almost twice as long as autosome 1. Suortti’s (1971) material consists of first meiotic metaphases obtained by either sectioning or squashing, and is not clear enough to give an accurate assessment of the karyotype.

**Figure 2.**
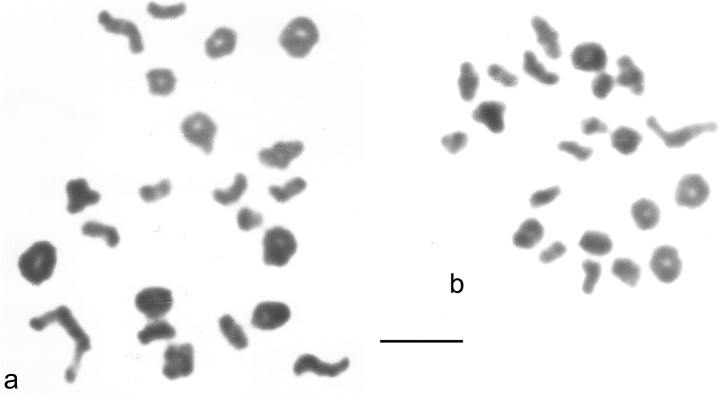
**a**, **b** *Agabus infuscatus* testis, first metaphase of meiosis. Bar = 5 μm.

*Agabus infuscatus* Aubé, 1838. Figs 1q, 2 (♂). Published information: none. 2n = 42 + neo XY. The autosomes are nearly all either metacentric or submetacentric, but pairs 3 and 17 are subacrocentric. The RCLs of the autosomes range from about 7.9–2.9, and there is a fairly even size decrease along the karyotype, though with slightly sharper decreases between pairs 1 (RCL about 7.9) and 2 (RCL about 6.9), 11 (RCL about 4.3) and 12 (RCL about 3.6), and pairs 18 (RCL about 3.6) and 19 (RCL about 2.9). The subacrocentric X-chromosome (RCL about 7.2) has a distinct gap in its long arm and the Y chromosome, also subacrocentric, is smaller, RCL about 4.6, and matches the X chromosome minus the terminal section of its long arm. This is typical of a neo-XY system where the X chromosome fuses with an autosome to give neo-X, and the same autosome without the X fused to it becomes the neo-Y chromosome. First metaphase of meiosis (Fig. 2) shows 22 bivalents with no suggestion of a B-chromosome behaving differently from the others. Although it is not possible to identify the neo-XY the behaviour of the chromosomes is entirely consistent with a neo-XY system.

### Subgenus *Gaurodytes* C. G. Thomson, 1859 The *Agabus bipustulatus* group

RCL data for this group are given in Table 2.

**Table 2.** *Agabus bipustulatus* group species, Relative Chromosome Length. Mean, 95% confidence intervals, number of chromosomes measured.

**Chromosome**	***Agabus bipustulatus***	***Agabus solieri***	***Agabus nevadensis***	***Agabus wollastoni***	***Agabus melanarius***
1	9.11<br/> 8.07–10.14<br/> N = 14	8.86<br/> 6.69–11.03<br/> N = 14	8.25<br/> 6.41–10.09<br/> N = 10	8.56<br/> 6.66–10.46<br/> N = 8	9.90<br/> 8.05–10.95<br/> N = 4
2	9.07<br/> 8.15–9.99<br/> N = 14	8.11<br/> 6.93–9.28<br/> N = 14	7.20<br/> 6.32–8.08<br/> N = 10	7.81<br/> 5.61–10.01<br/> N = 8	8.75<br/> 7.23–10.27<br/> N = 4
3	8.89<br/> 8.09–9.70<br/> N = 14	8.00<br/> 6.37–9.63<br/> N = 14	6.85<br/> 6.13–7.57<br/> N = 10	7.25<br/> 4.99–9.51<br/> N = 8	8.75<br/> 7.23–10.27<br/> N = 4
4	8.29<br/> 7.33–9.24<br/> N = 14	7.29<br/> 6.16–8.42<br/> N = 14	6.50<br/> 5.87–7.13<br/> N = 10	6.63<br/> 5.20–8.05<br/> N = 8	7.87<br/> 7.11–8.64<br/> N = 4
5	7.93<br/> 7.20–8.65<br/> N = 14	7.00<br/> 5.83–8.17<br/> N = 14	5.75<br/> 4.89–6.61<br/> N = 10	6.56<br/> 4.88–8.24<br/> N = 8	7.50<br/> 6.58–8.42<br/> N = 4
6	7.54<br/> 6.82–8.26<br/> N = 14	6.71<br/> 5.54–7.88<br/> N = 14	5.40<br/> 4.59–6.21<br/> N = 10	6.19<br/> 4.92–7.45<br/> N = 8	6.50<br/> 5.58–7.42<br/> N = 4
7	6.79<br/> 5.86–7.71<br/> N = 14	6.07<br/> 4.73–7.42<br/> N = 14	5.15<br/> 4.29–6.01<br/> N = 10	6.00<br/> 4.14–7.86<br/> N = 8	7.25<br/> 6.22–8.28<br/> N = 4
8	6.54<br/> 5.63–7.44<br/> N = 14	5.96<br/> 4.75–7.18<br/> N = 14	4.90<br/> 4.19–5.61<br/> N = 10	5.50<br/> 4.03–6.97<br/> N = 8	6.50<br/> 5.58–7.42<br/> N = 4
9	6.25<br/> 5.66–6.84<br/> N = 14	5.68<br/> 4.64–6.71<br/> N = 14	4.70<br/> 3.94–5.46<br/> N = 10	5.44<br/> 3.87–7.01<br/> N = 8	6.88<br/> 6.48–7.27<br/> N = 4
10	6.25<br/> 5.68–6.82<br/> N = 14	5.39<br/> 4.40–6.38<br/> N = 14	4.35<br/> 3.60–5.11<br/> N = 10	4.94<br/> 3.50–6.38<br/> N = 8	6.63<br/> 5.86–7.39<br/> N = 4
11	5.71<br/> 5.21–6.22<br/> N = 14	5.25<br/> 4.25–6.25<br/> N = 14	4.20<br/> 3.66–4.74<br/> N = 10	4.63<br/> 3.35–5.90<br/> N = 8	6.63<br/> 5.86–7.39<br/> N = 4
12	5.75<br/> 5.23–6.27<br/> N = 14	5.29<br/> 4.39–6.18<br/> N = 14	3.85<br/> 3.26–4.44<br/> N = 10	4.69<br/> 3.51–5.87<br/> N = 8	6.38<br/> 5.18–7.57<br/> N = 4
13	5.71<br/> 5.28–6.15<br/> N = 14	4.93<br/> 4.05–5.81<br/> N = 14	3.95<br/> 3.64–4.26<br/> N = 10	4.31<br/> 3.11–5.51<br/> N = 8	6.00<br/> 4.16–7.84
14	5.86<br/> 5.31–6.41<br/> N = 14	4.89<br/> 4.07–5.71<br/> N = 14	3.75<br/> 3.36–4.14	3.94<br/> 2.79–5.09<br/> N = 8	6.38<br/> 5.37–7.38<br/> N = 4
15	5.39<br/> 4.91–5.88<br/> N = 14	5.07<br/> 4.18–5.96<br/> N = 14	3.45<br/> 2.96–3.94<br/> N = 10	3.75<br/> 2.83–4.67<br/> N = 8	6.00<br/> 5.74–6.26<br/> N = 4
16	5.04<br/> 4.34–5.73<br/> N = 14	4.54<br/> 3.66–5.41<br/> N = 14	3.55<br/> 3.09–4.01<br/> N = 10	3.44<br/> 2.59–4.29<br/> N = 8	5.63<br/> 4.86–6.39<br/> N = 4
17	4.79<br/> 4.22–5.35<br/> N = 14	4.29<br/> 3.46–5.11<br/> N = 14	3.33<br/> 3.00–3.67<br/> N = 9	3.25<br/> 2.28–4.22<br/> N = 8	5.00<br/> 4.74–5.26<br/> N = 4
18	4.25<br/> 3.77–4.73<br/> N = 14	4.04<br/> 3.21–4.86<br/> N = 14	3.05<br/> 2.62–3.48<br/> N = 10	3.13<br/> 2.11–4.14<br/> N = 8	5.50<br/> 4.58–6.42<br/> N = 4
19	4.21<br/> 3.84–4.58<br/> N = 14	3.93<br/> 3.26–4.60<br/> N = 14	2.70<br/> 2.19–3.21<br/> N = 10	2.63<br/> 1.89–3.36<br/> N = 8	4.88<br/> 4.48–5.27
20	3.75<br/> 3.36–4.14<br/> N = 14	3.36<br/> 2.71–4.00<br/> N = 14	2.50<br/> 2.16–2.84<br/> N = 10	2.38<br/> 1.41–2.54<br/> N = 8	4.13<br/> 3.72–4.52<br/> N = 4
21	3.25<br/> 2.77–3.73<br/> N = 14	2.54<br/> 2.04–3.04<br/> N = 14	2.05<br/> 1.62–2.48<br/> N = 10	1.87<br/> 1.21–2.54<br/> N = 8	2.75<br/> 1.95–3.55<br/> N = 4
X	11.43<br/> 8.95–13.91<br/> N = 7	8.86<br/> 5.69–12.02<br/> N = 7	7.83<br/> 6.40–9.27<br/> N = 5	8.25<br/> 5.75–10.75<br/> N = 6	8.33<br/> 3.16–13.50<br/> N = 3

**Figure 3.**
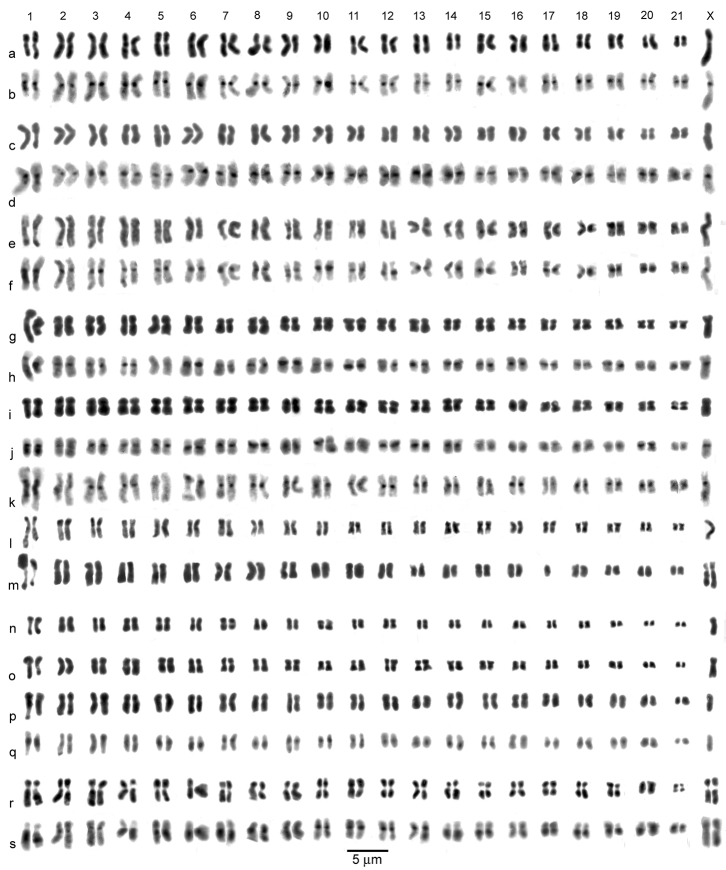
*Agabus (Gaurodytes)* part 1, the *Agabus bipustulatus* group, mitotic chromosomes arranged as karyotypes. **a**– **f** *Agabus bipustulatus*: **a–d** ♂, Inari, testis **a** plain **b** the same nucleus C-banded **c** plain **d** the same nucleus C-banded **e**, **f** ♂, Woolmer, mid-gut **e** plain **f** the same nucleus C-banded **g**– **k** *Agabus bipustulatus* var. *solieri*: **g, h** ♂, Moiry, testis **g** plain **h** the same nucleus C-banded **i–j, k** Illsee, ♂, testis **i** plain **j** the same nucleus C-banded **k** a different nucleus C-banded **l**, **m** *Agabus nevadensis*, Sierra Nevada, mid-gut, plain, **l** ♂, **m**♀ **n**, **o** *Agabus wollastoni*, ♂, Madeira, testis, plain; **p–s** *Agabus melanarius*: **p**, **q** ♂, testis **p** plain **q** the same nucleus C-banded **r**, **s** ♀, ovary, **r** plain, **s** the same nucleus C-banded. Bar = 5 μm.

*Agabus bipustulatus* (Linnaeus, 1767). Fig. 3a–f. Published information: 2n = 40 + Xy_p_ (Suortti 1971). (See comment on Suortti’s work under *Agabus sturmii*.) 2n = 42 + X0 (♂), 42 + XX (♀). The X chromosome is the longest in the nucleus, though its RCL value can overlap that of autosome 1 (Table 2). Autosome 1 is characterised by a secondary constriction in its long arm, frequently picked out by C-banding (Fig. 3b, d, f). The expansion or contraction of this constriction can drastically alter the apparent size of the chromosome (Fig. 3a, b). The longer chromosomes (pairs 1–10) are submetacentric, while the smaller ones are more or less metacentric. The X chromosome is submetacentric to subacrocentric. The variation in the apparent size of this chromosome in different nuclei can be striking–it is about twice as long as autosome 1 in Fig. 3a, b, but only slightly longer that autosome 1 in Fig. 3c, d. Since these nuclei are from the same beetle the difference must be the result of different degrees of condensation of the chromosome.

*Agabus bipustulatus* var. *solieri* Aubé, 1837. Fig. 3g–k. Published information: none. 2n = 42 + X0 (♂), 42 + XX (♀). All the preparations illustrated are from the Swiss Alps, and are chosen because good plain and C-banded preparations were obtained from the same nuclei. The nuclei shown in Fig. 3g–j are more condensed than the typical *Agabus bipustulatus* shown, but the one in Fig. 3k shows a comparable degree of condensation. These karyotypes show no obvious difference from those of typical *Agabus bipustulatus*. The dark area at the end of the X chromosome in Fig. 3k is where it overlapped one of the autosomes in the preparation. The extreme size difference between the two replicates of autosome 1 in Fig. 3g, h is very striking, but C-banding (Fig. 3h) shows that this size difference is entirely due to the degree of expansion of the secondary constriction.

*Agabus nevadensis* Håkan Lindberg, 1939. Fig. 3l, m. Published information: none. 2n = 42 + X0 (♂), 42 + XX (♀) The preparations are from old material in R. B. Angus’ archive, and no C-banding is available. The heavy short arm of one replicate of autosome 1 in Fig. 3m is the result of its lying on top of dark material. The sizes and shapes of these chromosomes show no detectable differences from those of *Agabus bipustulatus* and *Agabus bipustulatus* var. *solieri*.

*Agabus wollastoni* Sharp, 1882. Fig. 1n, o. Published information: none. 2n = 42 + X0 (♂). As with *Agabus nevadensis*, this is archive material and no C-banding is available. Only two karyotypes could be obtained, both from rather condensed nuclei, but the general arrangement of the chromosomes is very similar to, if not identical with, those of the species already discussed.

*Agabus melanarius* Aubé, 1837. Fig. 3p–s. Published information: none. 2n = 42 + X0 (♂), 42 + XX (♀). The general layout of the karyotype is very similar to those of the *Agabus bipustulatus* complex described above, but there appear to be more secondary constrictions. Thus in the female (Fig. 3s), where the C-banding is better displayed, secondary C-bands are clear in autosomes 1, 3, 6, 7 and 14, and even in the male (Fig. 3q) the secondary C-bands are clear in autosomes 1, 6 and 14.

### Other *Gaurodytes* species

**Figure 4.**
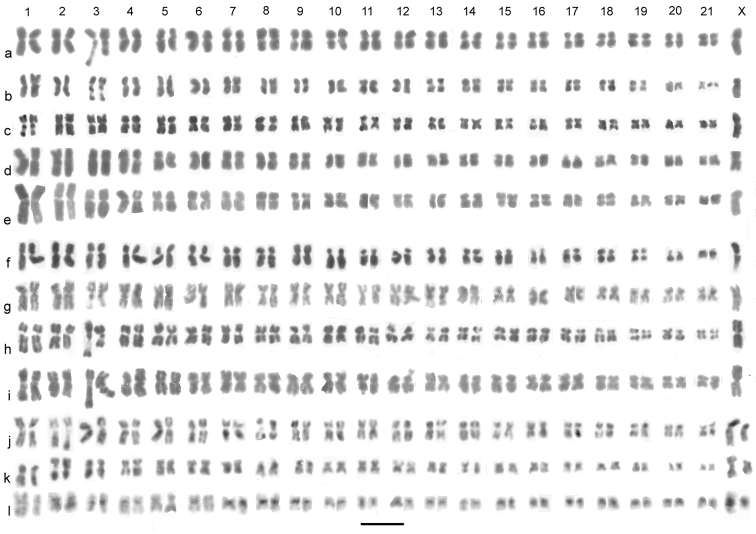
*Agabus (Gaurodytes)* part 2, mitotic chromosomes arranged as karyotypes. **a**, **b** *Agabus biguttatus*, ♂, mid-gut, plain: **a** El Noqra **b** Giara di Gesturi; **c** *Agabus binotatus*, ♂, mid-gut, plain **d** *Agabus affinis*, ♂, mid-gut, plain **e** *Agabus unguicularis*, ♂, mid-gut, plain **f** *Agabus ramblae*, ♂, Murcia, testis, plain **g** *Agabus conspersus*, ♂, mid-gut, plain **h**, **i** *Agabus nebulosus*, ♂, mid-gut, plain **h** Cuckmere **i** Tenerife **j–l** *Agabus adpressus*, ♂, mid-gut **j** plain **k, l** the same nucleus **k** plain **l** C-banded. Bar = 5 μm.

*Agabus biguttatus* (Olivier, 1795). Fig. 4a, b. Published information: 2n = 42 + X0 (♂), 22 + XX (♀) (Saleh Ahmed et al. 2000). The present material, from both Egypt and Sardinia, confirms the data of Saleh Ahmed et al. We have altered the position of the long chromosome with the secondary constriction from pair No. 3 to pair No. 1 as this matches the Sardinian specimen better, and there is sufficient variation in the RCL of this chromosome, due to opening of the secondary constriction to justify this move. The autosomes are all either metacentric or submetacentric with an even size decrease along the karyotype from RCL about 6 to about 3. The X chromosome has RCL about 6 and is more distinctly submetacentric than the larger autosomes, except of autosome 1 which has the secondary constriction. No C-banded preparation is available.

*Agabus binotatus* Aubé, 1837. Fig. 4c. Published information: none. 2n = 42 + X0 (♂). The karyotype of this species appears very similar to that of *Agabus biguttatus*, with a similar spread of RCLs. However, autosomes 14–21 are clearly less metacentric than in *Agabus biguttatus*, in some cases approaching subacrocentric. The X chromosome, RCL about 8.5, is clearly the largest in the nucleus, thus distinctly larger than in *Agabus biguttatus*.

*Agabus affinis* (Paykull, 1798). Fig. 4d. Published information: none. 2n = 42 + X0 (♂). The RCLs of the autosomes range from about 8–2.7, with an abrupt size decrease between pair 4 (RCL about 7.4) and pair 5 (RCL about 5.4), but otherwise with a gradual decrease. Most to the autosomes are either metacentric or submetacentric, but autosomes 12, 17, 20 and 21 are subacrocentric. The X chromosome is submetacentric, RCL about 6. No C-banded material is available.

*Agabus unguicularis* (Thomson, 1867). Fig. 4e. Published information: none. 2n = 42 + X0 (♂). The RCLs of the autosomes range from about 10–2.4. There is an abrupt size decrease between pairs 2 and 3 (RCLs about 9.4 and 7.6) and pairs 3 and 4 (RCL of pair 4 about 6.5), but apart from that the size decrease is fairly even. Most of the autosomes are metacentric or almost so, but a few are clearly submetacentric. The X chromosome, RCL about 6.5, is similar in size to autosome pair 4, but much more clearly submetacentric. No C-banded material is available.

*Agabus ramblae* Millan et Ribera, 2001. Fig. 4f. Published information: none. 2n = 42 + X0 (♂), 42 + XX (♀).The RCLs of the autosomes range from about 7–2.9, with a fairly even decrease in length along the karyotype. The autosomes are a mixture of metacentrics and submetacentrics (some at the extreme end of the range), with autosomes 10–12, 15, 16 and 20 subacrocentric. The X chromosome is about the same size as autosome 1, but more clearly submetacentric. No C-banded material is available.

*Agabus conspersus* (Marsham, 1802). Fig. 4g. Published information: 2n = 38 + XY (Yadav et al. 1984). 2n = 42 + X0 (♂). The RCLs of the autosomes range from about 6.1–3.6, with an even decrease in chromosome size along the karyotype. The autosomes are all either metacentric or submetacentric, and autosome 3 has a prominent secondary constriction in its long arm and autosome 15 has what appears to be a terminal NOR at the end of its short arm. The X chromosome, RCL about 5.6, is submetacentric and similar in size to autosomes 4–6. No C-banded material is available. This karyotype is clearly very different from that reported by Yadav et al. (1984). They report a number of nuclei supporting their conclusions, so the most likely explanation is that they were working with a different species. It may be noted that Marsham (1802) described *Agabus conspersus* from England so the material here may be regarded as true *Agabus conspersus*. Yadav et al. worked with Indian material.

*Agabus nebulosus* (Forster, 1771). Fig. 4h, i. Published information: none. 2n = 42 + X0 (♂), 42 + XX (♀). The general layout of the karyotype in terms of RCLs of the autosomes is very similar to that of *Agabus conspersus*. Autosome 3 has a similar secondary constriction in its long arm, but the small chromosome with the terminal apparent NOR is relatively larger than in *Agabus conspersus*, and is placed as pair 12 as against 15. The X chromosome, RCL about 7.3, appears relatively larger than that of *Agabus conspersus*, and is metacentric. The Tenerife specimen whose chromosomes are shown in Fig. 4i is of a form whose dark pronotal spots are absent or scarcely apparent, but the chromosomes clearly associate it with the British well-spotted *Agabus nebulosus* rather than *Agabus conspersus* which lacks the pronotal spots.

*Agabus adpressus* Aubé, 1837. Fig. 4j–l. Published information: none. 2n = 42 + XY (♂). The autosomes are all either metacentric or submetacentric, with RCLs ranging from about 7.2–3.1 and with an even decrease in size along the karyotype. Autosome 2 has a secondary constriction in its long arm and autosome 8 has one in its short arm. The X chromosome is submetacentric (almost metacentric), about as long as autosome 1. The Y chromosome, RCL about 5, looks like the X chromosome with most of one arm missing. C-banding (Fig. 4l) shows considerable variation in the centromeric C-bands of the autosomes. Autosome 1 lacks any C-band, 2 and 3 have strong C-bands and 4 has a weak one. Autosome 5 lacks a C-band and that on autosome 6 is very weak. Autosomes 7–9 have strong centromeric C-bands and 10–13 have weaker ones. Pair 14 has very weak bands. Pairs 15–21 have strong C-bands. The secondary constriction of autosome 2 shows as a C-band, but that of autosome 8 appears to be merged with the strong centromeric C-band. The sex chromosomes both have very large strong centromeric C-bands, which is a powerful piece of evidence that this is a neo-XY system rather than an X0 system and a B-chromosome. Unfortunately no meiotic preparation is available.

### Colymbetinae Erichson, 1837 *Colymbetes* Clairville, 1806

**Figure 5.**
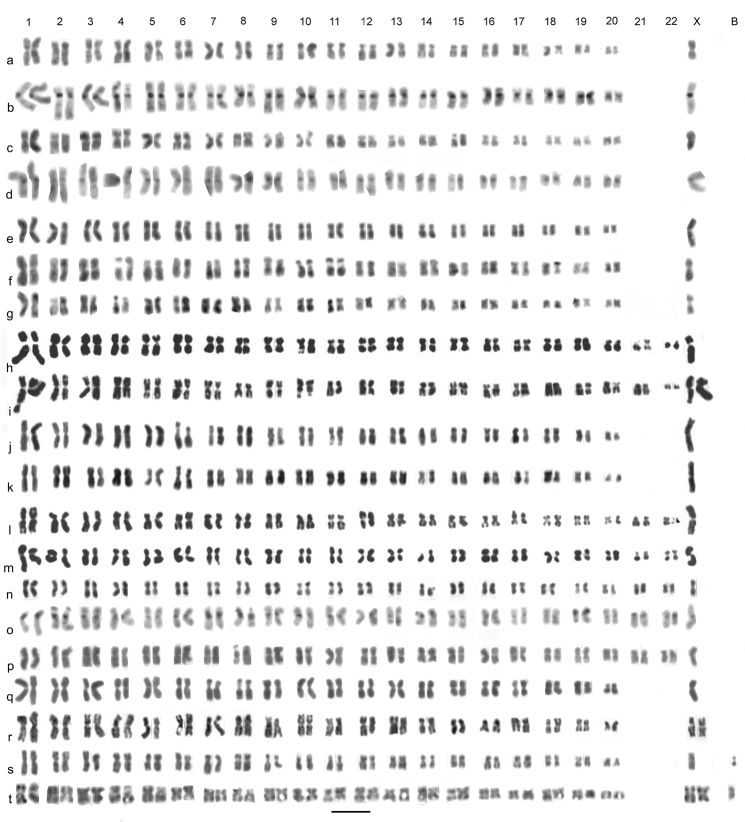
*Colymbetes* (**a–g**) and *Rhantus* (**h–t**), mitotic chromosomes arranged as karyotypes. **a**, **b** *Colymbetes fuscus*, ♂, mid-gut **a** Pinail, plain **b** Wisley, C-banded **c**, **d** *Colymbetes paykulli*, ♂, midgut, Mullsjö **c** plain **d** C-banded **e** *Colymbetes piceus*, ♂, Egypt, mid-gut, plain **f**, **g** *Colymbetes striatus*, ♂, mid-gut, plain **h**, **i** *Rhantus grapii*, ♂, mid-gut, plain **j**, **k** *Rhantus exsoletus*, ♂, mid-gut, plain **j** Studland Heath **k** Gayton Thorpe Common **l**, **m** *Rhantus frontalis*, ♂, plain **l** mid-gut, Gayton Thorpe Common **m** testis, Thompson Common **n–p** *Rhantus suturalis*, ♂ **n**, **o** mid-gut, Staines Moor **n** plain **o** C-banded **p** testis, Ras Az Zawr, Kuwait, plain **q–t** *Rhantus suturellus*, plain **q**♂, testis, Pinail **r–t** Studland Heath **r**♀ mid-gut **s**♂ mid-gut with 1 Bchromosome **t**♀ mid-gut with 1 B-chromosome. Bar = 5 μm.

*Colymbetes fuscus* (Linnaeus, 1758). Fig. 5a, b. Published information: 2n = 35–37 (♀) (Günthert 1910). 2n = 40 + X0 (♂). The RCLs of the autosomes range from about 7.8–2.1, with an even decrease in chromosome size along the karyotype. Autosomes 2, 9, 11, 12, 14 and 15 are subacrocentric, while the remainder are more or less metacentric. The X chromosome, RCL about 5.7, is metacentric, similar in size to autosome 8. All the chromosomes have distinct centromeric C-bands and autosome 4 has a fainter band, possibly a secondary constriction, in its short arm.

*Colymbetes paykulli* Erichson, 1837. Fig. 5c, d. Published information: 18 pairs including Xy_p_? (Suortti 1971). 2n = 40 + X0 (♂). The RCLs of the autosomes range from about 8.4–3.2, with an even decrease in chromosome size along the karyotype. The X chromosome, RCL about 6.8, is metacentric. Autosomes 2 and 12–16 are borderline submetacentric-subacrocentric, while autosome 17 is more clearly submetacentric. The general arrangement appears very similar to that of *Colymbetes fuscus* but the centromeric C-bands appear less bold (perhaps a preparation artefact). The unreliability of Suortti’s data has been mentioned under *Agabus sturmii* and *Agabus bipustulatus*.

*Colymbetes piceus* Klug, 1834. Fig. 5e. Published information: 2n = 40 + X0 (♂), 40 + XX (♀) (Saleh Ahmed et al. 2000). The karyotype shown in Fig. 5e is the one published by Saleh Ahmed et al. and is included for comparison with the other species. The RCLs of the autosomes range from about 8.2–1.9, with a fairly even decrease in chromosome size along the karyotype. Autosomes 2, 8, 12, 14 -17 and 19 are submetacentric, pair 9 is subacrocentric, and the remainder are metacentric. Autosomes 6 and 7 have secondary constrictions in their short arms. The X-chromosome, RCL about 8.2, is similar in size to autosome 1, but is less evenly metacentric. No C-banded material is available.

*Colymbetes striatus* (Linnaeus, 1758). Fig. 5f, g. Published information: 19 - 21 pairs + Xy_p_? (Suortti 1971). 2n = 40 + X0 (♂). The RCLs of the autosomes range from about 8–2.7, with a more noticeable decrease in length between autosomes 1 and 2 (RCL about 6.1) than in the other species, but otherwise with a fairly even decrease in chromosome length along the karyotype. Autosomes 2, 3, 5, 7 and 9 are submetacentric, but the others are more or less metacentric. Autosomes 4, 8 and 9 have secondary constriction in their short arms. The X chromosome, RCL about 9, is similar in length to autosomes 2–4, more nearly metacentric than pairs 2 and 3, but less so than pair 4. No C-banded material is available. For Suortti’s data, see comment under *Colymbetes paykulli*.

### *Rhantus* Dejean, 1833 Subgenus *Nartus* Zaitsev, 1907

*Rhantus grapii* (Gyllenhal, 1808). Fig. 5h, i. Published information: none. 2n = 44 + X0 (♂), 44 + XX (♀). The RCLs of the autosomes range from about 9.6–2.5, with an even decrease in chromosome length along the karyotype, apart from sharp decreases in size between pairs 1 and 2 (RCL about 7) and between pair 21 (RCL about 5.5) and pair 22 (RCL about 1.2). Most of the autosomes are more or less metacentric, but pairs 4, 5, 7, 10–12, 14, 16–18 and 20 are clearly submetacentric and pairs 21 and 22 are subacrocentric. Autosome 9 has a secondary constriction towards the end of its long arm. The X chromosome, RCL about 7.7, is the second to longest in the nucleus and is submetacentric. No C-banded material is available.

### Subgenus *Rhantus* s. str.

*Rhantus exsoletus* (Forster, 1771). Fig. 5j, k. Published information: 20 pairs + Xy_p_ (Suortti 1971). 2n = 40 + X0 (♂). The RCLs of the autosomes range from about 8–2.2, with a fairly even decrease in chromosome length along the karyotype. Autosome pairs 4, 7, 8, 10, 11, 15–17, 19 and 20 are clearly submetacentric, with pairs 9, 12 and 14 more or less subacrocentric. The remaining seven pairs are more or less metacentric. Pair 6 has a secondary constriction on its short arm. The X chromosome, RCL about 9, is the longest in the nucleus. No C-banded material is available.

*Rhantus frontalis* (Marsham, 1802). Fig. 5l, m. Published information (as *Rhantus notatus* F.): 22 pairs including Xy_p_ (Suortti 1971). 2n = 44 + X0 (♂). The RCLs of the autosomes range from about 8.3–2.7 with a sharper decrease in length between pair 1 and pair 2 (RCL about 6.4) but otherwise an even decrease in chromosome length along the karyotype. Autosomes 2–5, 7, 9–11, 14, 15 and 21 are submetacentric while the rest are more or less metacentric. Autosomes 2, 3 and 17 have secondary constrictions in their short arms. The X chromosome, RCL about 9.1, is the longest in the nucleus. No C-banded material is available.

*Rhantus suturalis* (Macleay, 1825). Fig. 5n–p. Published information: none. 2n = 44 + X0 (♂). The RCLs of the autosomes range from about 6.1–3.5. The rate of decrease along the karyotype is very even with many of the adjacent pairs appearing more or less the same size. Most of the autosomes are more or less metacentric but pairs 13, 14 and 22 are clearly submetacentric and pair 20 is subacrocentric. C-banding (Fig. 5o) shows all the chromosomes with centromeric C-bands, of varying strengths. Pairs 1, 8 and 14 have secondary constrictions on their short arms. The X chromosome, RCL about 5.8, is metacentric with a rather weak centromeric C-band. The Kuwaiti material (Fig. 5p) shows no differences from the British.

*Rhantus suturellus* (Harris, 1828). Fig. 5q–t. Published information: none. 2n = 40 + X0 (♂), 40 + XX (♀), sometimes with 1 B-chromosome. The RCLs of the autosomes range from about 7–2.7, with a fairly even decrease in chromosome length along the karyotype. Autosomes 5, 7, 9, 11, 12, 15 -17, 19 and 20 are clearly submetacentric, with the remainder more or less metacentric. Pairs 4, 8 and 9 have secondary constrictions in their short arms. The X chromosome, RCL about 5.3, is metacentric and similar to chromosomes 4–8. No C-banded material is available. This karyotype is unusual in having a B-chromosome, a small metacentric, RCL about 3, which has so far been found in Studland Heath material. The first Studland Heath material, in 1993, comprised a male with a B-chromosome and a female without one, giving the impression that this species had an XY sex chromosome system. However, the 2000 material, a mail from Pinail lacking the B-chromosome and a female from Studland Heath with the B-chromosome, revealed the true nature of the situation.

## Discussion

In considering the data presented here, two aspects are of particular note: the extent to which the different genera have characteristic karyotypes and details of any deviations from generic karyotypes; and the extent to which the karyotypes of related species show clear differences.

In *Agabus* 18 of the 20 species reported have a karyotype involving 21 pairs of autosomes and sex chromosomes which are X0 (♂) and XX (♀), but the remaining 2, *Agabus infuscatus* and *Agabus adpressus*, have 21 pairs of autosomes and sex chromosomes which are XY (♂) and XX (♀). These two species are not closely related (they are placed in different subgenera), but appear to have evolved similar neo-XY sex chromosomes. What makes this particularly surprising is that, since the development of a neo-XY system involves fusion of the original X chromosome with an autosome, there should be an initial reduction by one in the number of pairs of autosomes. However, both the species involved here show no such reduction, so have presumably undergone fission of one autosome to give two and hence restore the original number. It may be noted that Yadav et al. (1984) describe their “*Agabus conspersus*” as having 38 autosomes (19 pairs) and XY sex chromosomes. Assuming their chromosome data are correct and they are working with an *Agabus* species, this one has a reduced number of autosomes as well as an XY system.

Among the *Agabus* species reported here, there are two groups of particularly close relatives, *Agabus congener*, *lapponicus* and *thomsoni*, and the *Agabus bipustulatus* group. *Agabus congener* and *lapponicus* show no interspecific chromosomal differences despite a good number of high-quality preparations. *Agabus thomsoni* may show a slight difference in the RCL of the longest secondary constriction-bearing autosome, but more material would be needed to confirm this.

The *Agabus bipustulatus* group comprises *Agabus melanarius* and the *Agabus bipustulatus* complex within which the overriding impression from the present investigation is the extreme similarity between the karyotypes of the species. In the case of *Agabus bipustulatus* and *Agabus bipustulatus* var *solieri* this is not surprising as these are regarded as conspecific. The case of *Agabus nevadensis* is perhaps more interesting as this is currently regarded as a distinct species in spite of the lack of clear morphological characters to distinguish it from *Agabus bipustulatus*. The karyotype of *Agabus wollastoni* also shows no obvious difference from those of the other species, but in this case the species does have a very clear morphological character to distinguish it from *Agabus bipustulatus*–the inner anterior tarsal claw of the male is simple, not expanded to give the “scooped-out” appearance characteristic of *Agabus bipustulatus*, *solieri* and *nevadensis*. Only *Agabus melanarius*, not really a member of the *Agabus bipustulatus* complex, shows some karyotype differences, most clearly in the more extensive development of heterochromatic (C-banding) regions on the chromosomes. These findings may be considered in the light of the phylogenetic trees obtained by Drotz et al. (2010) from their studies of mitochondrial DNA of these beetles. Drotz et al. place the *Agabus bipustulatus* group as a complex within a slightly larger *Agabus tristis* Aubé group. Their Fig. 5 shows a strict consensus phylogenetic tree of the group. This figure is particularly interesting: *Agabus melanarius* is shown to be among the most isolated of the *Agabus tristis* group species, with it plus *Agabus tristis* placed as a sister taxon to all the rest combined. The remaining species, including *Agabus wollastoni*, comprise the *Agabus bipustulatus* complex, within which *Agabus wollastoni* is the first to come out, being placed as sister to all the others. It is at once apparent that the karyotypes of all these *Agabus bipustulatus* complex species are the ones showing no difference from one another. *Agabus melanarius* does show chromosomal differences, and it would be very interesting to know whether this is also true of *Agabus tristis*. However, this is a Nearctic and east Palaearctic species, not available for study here.

Examination of the material of *Agabus bipustulatus*, *solieri* and *nevadensis* included in their study shows how they came to their conclusions as to their taxonomic status. They are concerned with forms in which the primary reticulation (the fine meshes inside the larger elongate secondary meshes) is progressively reduced. These forms are referred to the varieties *dolomitanus* Scholz, 1935, *falcozi* Guignot, 1932, *kiesenwetteri* Seidlitz 1887 and *pyrenaeus* Fresneda and Hernando, 1989. The most striking thing is that these various *solieri* forms come out in a number of different places, often with ordinary *bipustulatus* from neighbouring areas. *Agabus nevadensis*, with its very restricted distribution, almost inevitably comes out in only one place, but very closely associated with a population of *solieri* (*kiesenwetteri*) from France. The claim of *Agabus nevadensis* to species status appears weak. The mitochondrial DNA separation is very slight, the karyotype appears identical with those of other *Agabus bipustulatus* forms, and the morphological characteristics are less clear than those of *solieri*.

The case of *Agabus wollastoni* is interesting. This species is isolated on Madeira and has had time to diverge from other *Agabus bipustulatus*, both in its mitochondrial DNA and also in its morphology–simple inner anterior tarsal claws of males, and generally larger size. Only the chromosomes show no difference.

The four species of *Colymbetes* share the same basic karyotype with 2n = 40 + X0 (♂), with the X chromosome a large more or less metacentric. There are minor differences in the RCL sequences between the species, which may or may not stand up to more detailed analysis if more material becomes available. Autosome 1 of *Colymbetes striatus* appears larger than in the other species.

The karyotypes of the *Rhantus* species are interesting in showing two different numbers, with 2n = 40 + X0 (♂) in *Rhantus exsoletus* and *Rhantus suturellus*, but 2n = 44 + X0 (♂) in the other species studied. Interestingly, this number difference does not reflect the subgeneric classification. The B-chromosome of *Rhantus suturellus* is interesting in that it could be confused with a neo-XY sex chromosome system comparable with that of *Agabus infuscatus* and *Agabus adpressus*.

The Kuwaiti material of *Rhantus suturalis* is interesting as it shows no differences from British material. Balke et al. (2009) demonstrated that this “supertramp” species almost certainly originated in the highlands of New Guinea from where it extended its range in two separate lineages, one southern going into Australia and New Zealand, and the other northern, going into Asia and Europe. Clearly Kuwaiti and European material belong to this northern lineage, but it is good to see the absence of chromosomal differences between specimens from these areas supporting the integrity of this species.
